# P152: Reduction of the prevalence of cholera by observation of hand hygiene

**DOI:** 10.1186/2047-2994-2-S1-P152

**Published:** 2013-06-20

**Authors:** T Koto, AT Ahoyo, J Akpovij, M Mensah, Fanou, O Binazon

**Affiliations:** 1 Health Ministry of Benin, Cotonou, Benin

## Objectives

Benin is endemic to epidemic area for diarrhea and cholrea in particular. The object is to present the result of the promotion campaign of sanitary measures which permit to reduce cholera spread in the studied area.

## Methods

The study was focused on two areas affected by cholera in September 2012. The sensitization (close communication) during three months, by focus group in the families, interviews with people, direct observation of behavior of affected people was performed from September to December 2012. Inspections at place selling road food was added to the different actions.

## Results

In an estimated population of 250 000 the handwashing increased from 23% to 65%, 79% of 2183 contaminated wells and 189 tanks were systematically treated. Of 506 infected people, we have noticed 276 cured patients and 02 deaths. The remained were in observation at the end of the study.

## Conclusion

Wells ‘treatment coupled by handwashing with water and soap permitted to reduce the frequency of cholera in two sanitary areas. An intensive approach communication‘s work and health education is necessary to insert hands washing in our best practices.

## Disclosure of interest

None declared

